# Epidemiology and mortality of pelvic and femur fractures—a nationwide register study of 417,840 fractures in Sweden across 16 years: diverging trends for potentially lethal fractures

**DOI:** 10.1080/17453674.2021.1878329

**Published:** 2021-01-28

**Authors:** Natalie Lundin, Tuomas T Huttunen, Anders Enocson, Alejandro I Marcano, Li Felländer-Tsai, Hans E Berg

**Affiliations:** a Department of Molecular Medicine and Surgery, Karolinska Institute, Karolinska University Hospital, Stockholm, Sweden;; b Division of Orthopedics and Biotechnology, Department of Clinical Science, Intervention and Technology, Karolinska Institute, Karolinska University Hospital, Stockholm, Sweden;;; c Faculty of Medicine and Health Technology, Tampere University, Tampere University Hospital, Tampere, Finland;; d Department of Emergency, Anesthesia and Pain Medicine, Tampere University Hospital, Tampere, Finland

## Abstract

Background and purpose — Fractures of the pelvis and femur are serious and potentially lethal injuries affecting primarily older, but also younger individuals. Long-term trends on incidence rates and mortality might diverge for these fractures, and few studies compare trends within a complete adult population. We investigated and compared incidence and mortality rates of pelvic, hip, femur shaft, and distal femur fractures in the Swedish adult population.

Patients and methods — We analyzed data on all adult patients ≥ 18 years in Sweden with a pelvic, hip, femur shaft, or distal femur fracture, through the Swedish National Patient Register. The studied variables were fracture type, age, sex, and 1-year mortality.

Results — While incidence rates for hip fracture decreased by 18% (from 280 to 229 per 10^5^ person-years) from 2001 to 2016, incidence rates for pelvic fracture increased by 25% (from 64 to 80 per 10^5^ person-years). Incidence rates for femur shaft and distal femur fracture remained stable at rates of 15 and 13 per 10^5^ person-years respectively. 1-year mortality after hip fracture was 25%, i.e., higher than for pelvic, femur shaft, and distal femur fracture where mortality rates were 20–21%. Females had an almost 30% lower risk of death within 1 year after hip fracture compared with males.

Interpretation — Trends on fracture incidence for pelvic and femur fractures diverged considerably in Sweden between 2001 and 2016. While incidence rates for femur fractures (hip, femur shaft, and distal femur) decreased or remained constant during the studied years, pelvic fracture incidence increased. Mortality rates were different between the fractures, with the highest mortality among patients with hip fracture.

Pelvic and femur fractures are potentially lethal to both young and elderly patients (Deakin et al. 2007). The younger multi-traumatized patient risks fatal bleeding or other simultaneous mortal injuries after high-energy trauma (Enninghorst et al. 2013). Frail elderly patients exhibit high mortality during the first months after simple falls (Reito et al. 2019). While proximal femur (hip) fractures among the elderly are well studied with respect to incidence and mortality, pelvic and non-hip femur fractures are less well described, and comparisons within a complete population are lacking.

Hip fracture incidence has after many years of steady increase actually stabilized in several Western populations, and even decreased during the last decades, as especially evident in Scandinavia (Cooper et al. 2011, Rosengren et al. 2017, Kannus et al. 2018). Pelvic fractures seem instead to maintain an increasing incidence (Kannus et al. 2015, Melhem et al. 2020). While less frequent than hip and pelvic fractures, it has been suggested that shaft and distal femur fractures are increasing (Ng et al. 2012).

1-year mortality after hip fracture has globally been described to be between 18% and 27%, trending downwards (Downey et al. 2019). Mortality data on pelvic and distal femur fractures points at similar or higher levels, while data on femur shaft fractures is scarce (Streubel et al. 2011, Moloney et al. 2016, Reito et al. 2019). Little has been published regarding mortality for pelvic and femur fractures within whole populations, and to our knowledge no study has compared the incidence and mortality rates within a complete national population.

We investigated and compared the incidence and mortality rates of pelvic, hip, femur shaft, and distal femur fractures in the Swedish adult population over time, including age and sex distribution.

## Patients and methods

We used the Swedish National Patient Register (NPR) to find data on all healthcare visits with relevant diagnoses between 2001 and 2016. The NPR was established by the Swedish National Board of Health and Welfare in 1964 and has a high coverage of both in- and outpatients (Ludvigsson et al. 2011). The register is based on admission/discharge from caregivers, and includes data on personal identity number, age, sex, diagnoses and surgical procedures. Diagnostic codes according to ICD-10 are used by the register.

We performed a search for pelvic fractures, including acetabulum (S32.1, S32.3, S32.4, S32.5, S32.7, S32.8), hip fractures (S72.0, S72.1, S72.2), femur shaft fractures (S72.3), and distal femur fractures (S72.4) from 2001–2016. We included all persons ≥ 18 years with a valid personal identity number and with any of the above fracture codes. Collected variables included age, sex, and diagnosis. Incidence rate was calculated as person-time incidence rate and was expressed as rate per 10^5^ person-years. Statistics regarding the Swedish population were found via the open access register from Statistics Sweden (www.scb.se), and the reported population on July 1st each year was used as a representation for the whole year.

We used the Swedish Cause of Death Register to investigate 1-year mortality in the fracture cohorts. The register contains all deaths registered in Sweden. The national registration number, a unique identifier assigned to all Swedish citizens, allows linkage of data between registers.

The 1st admission for a fracture was regarded as the incident case, and subsequent visits were counted anew if the visit contained another fracture code or was encountered beyond 12 months from the 1st visit. This means patients could be included more than once if another fracture type occurred at any time, or the same fracture type occurred after a time frame of 1 year. Patients with concomitant fractures at the same time were included in several fracture groups according to fracture.

### Statistics

Data was extracted from a pseudonymized SAS database (SAS Institute, Cary, NC, USA) and statistical analysis was done using R version 4.0.0 (R Centre for Statistical Computing, Vienna, Austria). Data was stored in accordance with current GDPR regulations. Statistical testing of differences in mean age was done using a 1-way ANOVA test. The average change in the number of fractures was estimated using Poisson regression.

### Ethics, funding, and potential conflicts of interest

The study was approved by the regional ethics committee (reference numbers: 2013/581-31/5 and 2016-2251-32) and was performed according to the standards of the 1964 Declaration of Helsinki. Funding was received from the Regional Agreement on Medical Training and Clinical Research between Stockholm County Council and the Karolinska Institute (ALF). None of the authors report any conflict of interest.

## Results

The total number of pelvic and femur fractures during the study period was 417,840. The 71% hip fractures represented the largest fracture group in this material, followed by the pelvic fractures at 21%. The femur shaft and distal femur fractures represented 4.3% and 3.6% respectively of all fractures (Table 1). The Swedish adult population ≥ 18 years increased by 14% during the studied years (Statistics Sweden). Mean age was between 68 and 80 years in the fracture cohorts with lowest mean age among femur shaft fractures (68 years, SD 23), and highest among hip fractures (80 years, SD 11). Differences in mean age were statistically significant (p < 0.001). Sex distribution showed a female dominance among all fracture cohorts (60–73%), with lowest proportion of females among femur shaft fractures (Table 2).

**Table 1.** Number of pelvic and femur fractures in patients ≥ 18 years in Sweden between 2001 and 2016

Year	Pelvic	Hip	Femur shaft	Distal femur	Total
2001	4,472	19,549	1,104	903	26,028
2002	4,493	18,930	1,111	901	25,435
2003	4,287	19,029	1,035	883	25,234
2004	4,482	18,987	1,111	850	25,430
2005	4,680	18,649	1,080	910	25,319
2006	5,171	18,979	1,131	925	26,206
2007	5,401	18,611	1,152	983	26,147
2008	5,479	19,088	1,176	972	26,715
2009	5,592	18,550	1,200	1,005	26,347
2010	6,021	18,745	1,209	947	26,922
2011	6,046	18,665	1,248	993	26,952
2012	5,977	17,926	1,060	895	25,858
2013	6,203	18,128	1,098	943	26,372
2014	6,117	17,635	1,056	890	25,698
2015	6,554	17,922	1,192	966	26,634
2016	6,333	18,098	1,160	952	26,543
Total	87,308	297,491	18,123	14,918	417,840

**Table 2.** Baseline patient characteristics for pelvic, hip, femur shaft, and distal femur fracture

	Pelvic	Hip	Femur shaft	Distal femur
Factor	n = 87,308	n = 297,491	n = 18,123	n = 14,918
Sex, n (%)				
Male	25,495 (29)	95,295 (32)	7,304 (40)	3,994 (27)
Female	61,813 (71)	202,196 (68)	10,819 (60)	10,924 (73)
Mean age (SD)				
Total	75 (18)	80 (11)	68 (23)	71 (20)
Male	68 (21)	78 (13)	57 (25)	56 (22)
Female	78 (16)	82 (10)	76 (18)	77 (16)

### Pelvic fracture incidence

**Figure 1.**
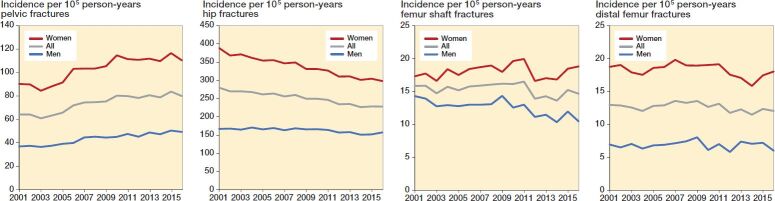
Yearly total incidence rate of all pelvic fractures, hip fractures, femur shaft fractures, and distal femur fractures in patients ≥ 18 years in Sweden 2001–2016.

**Figure 2.**
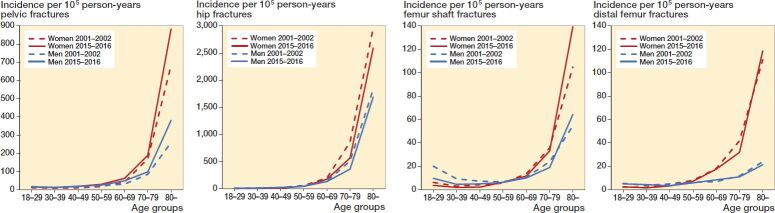
Mean incidence rate of all pelvic fractures, hip fractures, femur shaft fractures, and distal femur fractures in patients ≥ 18 years in Sweden 2001–2002 and 2015–2016 per age group.

The incidence of pelvic fractures rose by 25% during the study period, from 64 to 80 per 10^5^ person-years (Figure 1). The average annual increase in the number of fractures was 2.9%. The increase in incidence of pelvic fractures was seen in both males and females, and mainly in the oldest population (Figures 1 and 2). The incidence of pelvic fractures for younger patients (aged 18–49) was low compared with the older age groups, between 11 and 17 per 10^5^ person years. The incidence of younger females aged 18–49 increased during the study period to the same level as that of the males (Table 3).

**Table 3.** Mean incidence rate per 10^5^ person-years in 2001–2002 and 2015–2016 for adults aged 18–49 years

	Men		Women	
Fracture site	2001–2002	2015–2016	2001–2002	2015–2016
Pelvic	16	16	11	17
Hip	13	12	6.5	5.8
Femur shaft	12	6.6	4.4	2.6
Distal femur	4.6	4.2	2.9	2.3

### Hip fracture incidence

The incidence of hip fractures decreased by 18% from 280 to 229 per 10^5^ person-years (Figure 1). The average annual decrease in the number of fractures was 0.5%. The decrease in incidence was mainly due to a gradual decrease in female incidence from 389 to 299 per 10^5^ person-years. The incidence of hip fractures in males was fairly steady and decreased only slightly from 168 to 158 per 10^5^ person-years in 2001–2016 (Figure 1). Hip fracture incidence increased markedly with age (Figure 2). Hip fractures were uncommon in young adults (18–49 years), at between 5.8 and 13 per 10^5^ person-years, nevertheless about twice as frequent in males compared with females but showing no evident changes over the study period (Table 3).

### Femur shaft fracture incidence

The overall incidence rate was fairly stable at around 15 per 10^5^ person-years during the studied years (Figure 1). The average annual increase in the number of fractures was 0.3%. Incidence was highest among people aged ≥ 80 years (Figure 2). Femur shaft fractures were more than twice as common in younger males as in females (Table 3). A decrease was seen among males aged 18–49, where the incidence rate was reduced by almost half during the study period (Table 3).

### Distal femur fracture incidence

The overall incidence rate remained stable at around 13 per 10^5^ person-years during the study period (Figure 1). The average annual increase in number of fractures was 0.4%. Distal femur fracture incidence increased markedly with age, especially in females (Figure 2). Females aged ≥ 80 years had an almost 5-fold incidence of distal femur fracture compared with males in the same age group (Figure 2d), which corresponds to the largest sex difference among the studied fractures. Distal femur fractures among young individuals were rare, at ≤ 5 fractures per 10^5^ person-years (Table 3).

### Mortality

**Figure 3.**
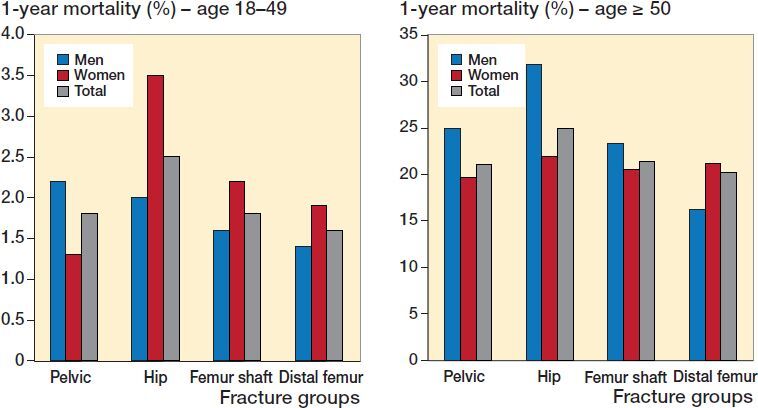
1-year mortality after pelvic, hip, femur shaft, and distal femur fractures in patients aged 18–49 and ≥ 50 years in Sweden between 2001 and 2016.

Unadjusted mortality within 1 year after pelvic, hip, femur shaft, or distal femur fracture was low among the younger population aged 18–49, between 1.3% and 3.5%. (Figure 3). In the population ≥ 50 years, 1-year mortality was highest after hip fracture at 25%. 1-year mortality for pelvic fracture was 21% and for femur shaft fracture and distal femur fracture this was 21% and 20% respectively among adults 50 years and older (Table 4). Sex differences in mortality in the older population were mainly seen among hip fracture patients (Figure 3). Males displayed both the highest (32% for hip fractures) and the lowest (16% for distal femur fractures) 1-year mortality within the cohort.

**Table 4.** 1-year mortality in percentage for pelvic, hip, femur shaft, and distal femur fractures in patients ≥ 18 years in Sweden between 2001 and 2016

Sex and age	Pelvic	Hip	Femur shaft	Distal femur
Males 18–49	2.2	2.0	1.6	1.4
Females 18–49	1.3	3.5	2.2	1.9
Total 18–49	1.8	2.5	1.8	1.6
Males ≥ 50	25	32	23	16
Females ≥ 50	20	22	21	21
Total ≥ 50	21	25	21	20

## Discussion

Our main finding was the diverging incidence trends between pelvic and hip fractures. While hip fracture incidence decreased by 18%, the incidence of pelvic fractures increased by 25% in the Swedish adult population between 2001 and 2016. Femur shaft and distal femur fractures showed marginal overall changes. Pelvic, hip, and other femur fractures remained rare in the young and middle-aged population. 1-year mortality was highest among hip fractures (25%) while similar for the other fracture groups (20–21%); however, sex differences were considerable.

### Incidence

Our hip fracture data was consistent with previously reported trends showing decreasing incidence rates from around the year 2000 in several European countries (Lucas et al. 2017). Kannus et al. (2018) reported a declining incidence rate of hip fractures after 1997 in individuals ≥ 50 years, in the entire Finnish population. We found a similar decline, mainly due to decreasing incidence rates among females ≥ 70 years, rendering a decline in the total number of Swedish hip fractures of 7.4%, despite a 14% increase in population numbers between 2001 and 2016.

Pelvic fracture incidence increased markedly during the same years due to steady increases among both older males and females. Accordingly, pelvic fracture was the only fracture type to show a pronounced increase in absolute numbers during the study period (from 4,472 to 6,333; 42%). Other studies have also reported increasing incidences of pelvic fractures (Kannus et al. 2015, Melhem et al. 2020), but numbers are conflicting. In the French population between 2006 and 2016, a 69% increase in the total number of pelvic and acetabular fractures was reported (Melhem et al. 2020). The French study reported lower overall incidence rates within the entire population, including children and adolescents < 18 years.

Studies on incidence rates in large populations of patients with femur shaft fractures are somewhat limited. Our incidence rate of 15 per 10^5^ person-years in 2016 was similar to previously reported numbers (Court-Brown and Caesar 2006, Weiss et al. 2009, Enninghorst et al. 2013). Earlier studies suggest a dominance in young males sustaining femur shaft fractures, and a bimodal distribution with regards to age and sex (Court-Brown and Cesar 2006, Enninghorst et al. 2013). We found both male and female femur shaft fractures to be more frequent in the older population. The distribution was bimodal during the first study years, but this faded with time due to fewer young males sustaining the fracture. Comparisons between studies are complicated by the differences in age of studied populations, where some studies included children and consequently found a considerably lower mean age than in this study, and with a marked incidence peak among younger men (Weiss et al. 2009, Ng et al. 2012, Enninghorst et al. 2013).

The distal femur fracture (incidence rate of 13 per 10^5^ person-years), overall as common as the shaft fracture, showed all signs of an osteoporotic fracture regarding age and sex distribution, with incidence increasing in women older than 60, and a steep rise with age. Interestingly, male fracture incidences were maintained as low throughout life, and were less than 20% of the female incidence even among the very old. These results confirm earlier reported trends of the distal femur fracture (Elsoe et al. 2018).

As expected, the vast majority of all fractures occurred in older individuals and 68% of the patients were female, in agreement with recent studies on the epidemiology of pelvic and hip fractures (Cauley et al. 2014, Kannus et al. 2015, 2018), and underpinned by the fact that hip and pelvic fractures together constituted 92% (71% and 21% respectively) of all counted fractures in our material. The 50% or larger incidence rate of females compared with males ≥ 70 years encountering a pelvic or femur fracture found in our study emphasizes the burden of osteoporosis in the female population.

Pelvic and femur fractures in the younger population, aged 18–49, were rather uncommon, with an incidence of 2.3–17/10^5^ person-years, with the distal femur fracture being especially uncommon (2.3–4.6/10^5^ person-years). Our numbers confirm previously published rates for younger adults (Farr et al. 2017). While adding only marginally to total fracture numbers, relative changes are still relevant to each patient group. The most prominent change among younger adults was the fall in femur shaft fracture incidence among males by almost half. Pelvic fractures among young females, conversely, showed a 50% increase in incidence that resulted in an equal rate between sexes. These changes are not easily explained and merit further investigation.

### Mortality

Mortality among patients ≥ 50 years after hip fracture was higher (25%) than for pelvic, femur shaft, and distal femur fracture (20–21%). Females exhibited an almost 30% lower risk of death within 1 year after hip fracture compared with males. Hip fracture mortality in this study confirmed previous findings with regards to both the overall 1-year risk of death, and sex differences (Abrahamsen et al. 2009, Downey et al. 2019).

Mortality within the 1st year after pelvic fracture has with few exceptions been investigated among inpatients only. A Dutch study of pelvic fractures among inpatients ≥ 65 years found a 1-year mortality of 27% (Banierink et al. 2019), substantially higher than the 21% found in our study. A German study from 2017, including both in- and outpatients, found a 1-year mortality of 21% (Andrich et al. 2017), like our results.

Reports on long-term mortality after femur shaft fracture are scarce, especially among older patients. A recent Swedish study investigated mortality in femur shaft fractures among patients ≥ 65 years and found a 1-year mortality of 21%, as in our study (Wolf et al. 2020). A German study investigating high-energy femur shaft fractures among young and middle-aged adults reported an in-hospital mortality of 10% (Kobbe et al. 2013). These numbers are considerably higher than the 1.8% found in our study for adults aged 18–49 years.

Our 20% 1-year mortality after distal femur fracture in adults ≥ 50 years was somewhat lower than previously reported (25%; Streubel et al. 2011, Moloney et al. 2016). However, those studies investigated only surgically treated patients (≥ 60 years), which could explain their higher mortality rates.

Earlier comparisons of mortality rates between hip and non-hip femur fractures either found similar rates (Streubel et al. 2011) or higher mortality rates for hip fractures, as found in our study (Deakin et al. 2007, Abrahamsen et al. 2009, Wolf et al. 2020). A Finnish study compared 30- and 90-day, mortality rates between patients with hip and pelvic fracture and found no differences (Reito et al. 2019). It can only be speculated as to whether the long-term clinical development affects differently the 1-year compared with 30- and 90-day mortality. Additionally, the treatments of pelvic and femur fractures are highly different and might confound comparisons. Fractures surgically treated, like the vast majority of hip fractures, are of course at more overt medical risk. However, the 1-year mortality reflects the final outcome in these typically old and fragile patients, with a female dominance. In order to unveil the details of the striking high male mortality after hip fracture, or the lower mortality in males after distal femur fracture, future studies, stratified for age, sex, and also surgical procedures, are suggested.

### Strengths and limitations

The major strength of this study is the large number of included patients within a complete national cohort that allowed for detailed and unbiased comparisons between the different fractures. Moreover, the included patient population was unselected and thus any regional, ethnic, or socioeconomic bias was minimized. The main limitation is potential misclassification in the registry data. Still, the NPR data has been reported to be of high quality and any misclassification should most likely be accidental and therefore any correlation biased “toward the null.” Another limitation concerns lack of information on comorbidities and cause of death, which could not be retrieved from the NPR register. The absence of this data might influence the comparability of the patient cohorts.

The stratification in age and sex intervals of the whole population in each year allowed the unique comparison between different fracture sites among the very same individuals in Sweden for that year. Regarding the demographics of our population, we found the total male–female ratio to be stable throughout the study years, and the different age groups constituted a similar proportion of the entire population, with changes smaller than 3% increase or decrease across the 16 years. Moreover, incidence calculations by each year used the exact number of Swedish males or females in that year for comparison.

## Conclusion

Incidence of hip fractures decreased while incidence of pelvic fractures increased in the Swedish adult population between 2001 and 2016. Mortality within 1 year after fracture was higher for hip fracture patients compared with patients with fracture of the pelvis, femur shaft, or distal femur.

Abrahamsen B, van Staa T, Ariely R, et al. Excess mortality following hip fracture: a systematic epidemiological review. Osteoporos Int 2009; 20(10): 1633-50.
